# Supplementary material for: Functional and structural characterization of AtAbf43C: an exo-1,5-α-L-arabinofuranosidase from Acetivibrio thermocellus DSM1313
Source: Biochem J. 2025 Aug 18;482(16):1181–95. doi: 10.1042/BCJ20253186 (PMC12493170; doi:10.1042/BCJ20253186)
Supplement: Online supplementary figure 1 [file bcj-482-16-BCJ20253186-s001.pdf]

Supplementary Information For:

Functional and structural characterization of AtAbf43C: An exo-1,5-α-L-arabinofuranosidase from *Acetivibrio thermocellus* DSM1313

Authors: Joey L. Galindo [1], Philip D. Jeffrey [2], Angela Zhu [1], A. James Link [1,2,3,4,5], Jonathan M. Conway [1,2,4,5,6,#]

Author Affiliations:

[1] Department of Chemical and Biological Engineering, Princeton University, Princeton, NJ 08544, USA

[2] Department of Molecular Biology, Princeton University, Princeton, NJ 08544, USA

[3] Department of Chemistry, Princeton University, Princeton, NJ 08544, USA

[4] Omenn-Darling Bioengineering Institute, Princeton University, Princeton, NJ 08544, USA

[5]Andlinger Center for Energy and the Environment, Princeton University, Princeton, NJ 08544, USA

[6]High Meadows Environmental Institute, Princeton University, Princeton, NJ 08544, USA

#Corresponding Author: Jonathan M. Conway jmconway@princeton.edu

Figure S1. (a) Structural visualization of residues mutated in AtAbf43C in the arabinose-soaked structure (PDB: 9NXH). **(b)** Protein gel of expressed and purified wild-type and mutant AtAbf43C used in subsequent activity assays.

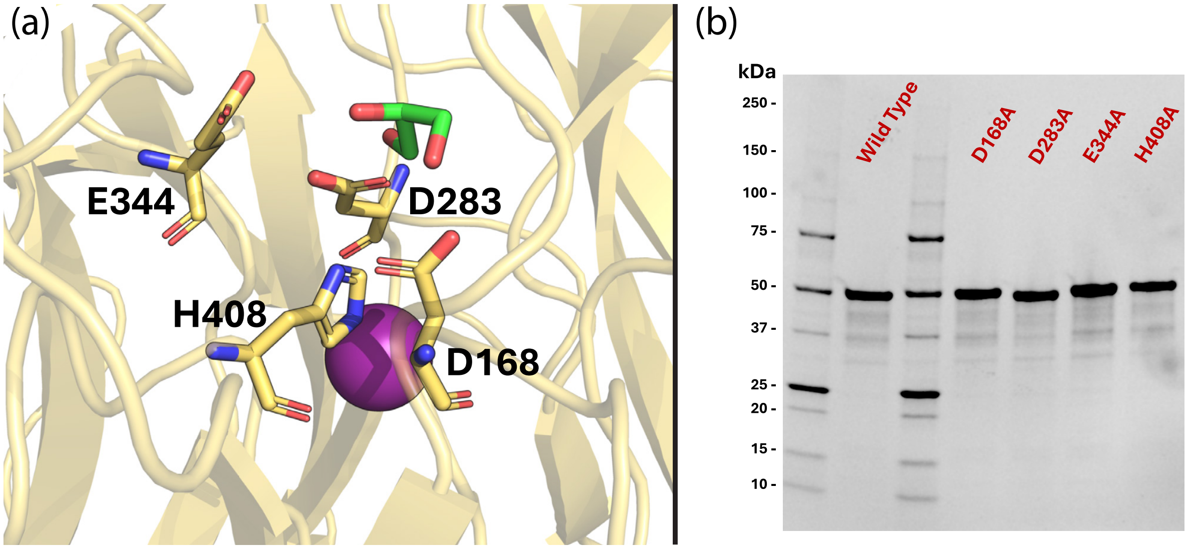

Figure S2. (a) Structural alignment of the CBM42 domain of arabinose soaked AtAbf43C structure (GH43 domain in yellow, CBM42 domain in red) with the structure of SaAraf43A complexed with arabinotriose (PDB Code: 3AKH) shown in light blue. **(b)** Alignment of residues that form hydrogen bonds with the arabinobiose molecule bound in α-pocket of the SaAraf43A CBM42 domain (light blue) with corresponding residues in the Atabf43C structure (red). **(c)** Alignment of residues that form hydrogen bonds with the arabinobiose molecule bound in ß-pocket of the SaAraf43A CBM42 domain (light blue) with corresponding arabinose bound residues in the Atabf43C structure (red). **(d)** Alignment of residues that form hydrogen bonds with the arabinose molecule bound in ɣ-pocket of the AtAbf43C CBM42 domain (red) with corresponding residues in the SaAraf43A structure (light blue). In **(a-d)** magnesium (Mg^{2+}) is shown as an orange sphere, sodium (Na^{+}) is shown as an orange sphere, chlorine (Cl^{-}) is shown as a green sphere, glycerol molecules are shown as green stick structures, and sugar molecules are shown as dark grey stick structures. Hydrogen bonds between residues and sugars the SaAraf43A structure are shown as dashed cyan lines, while hydrogen bonding in the AtAbf43C structure are shown as yellow dashes.

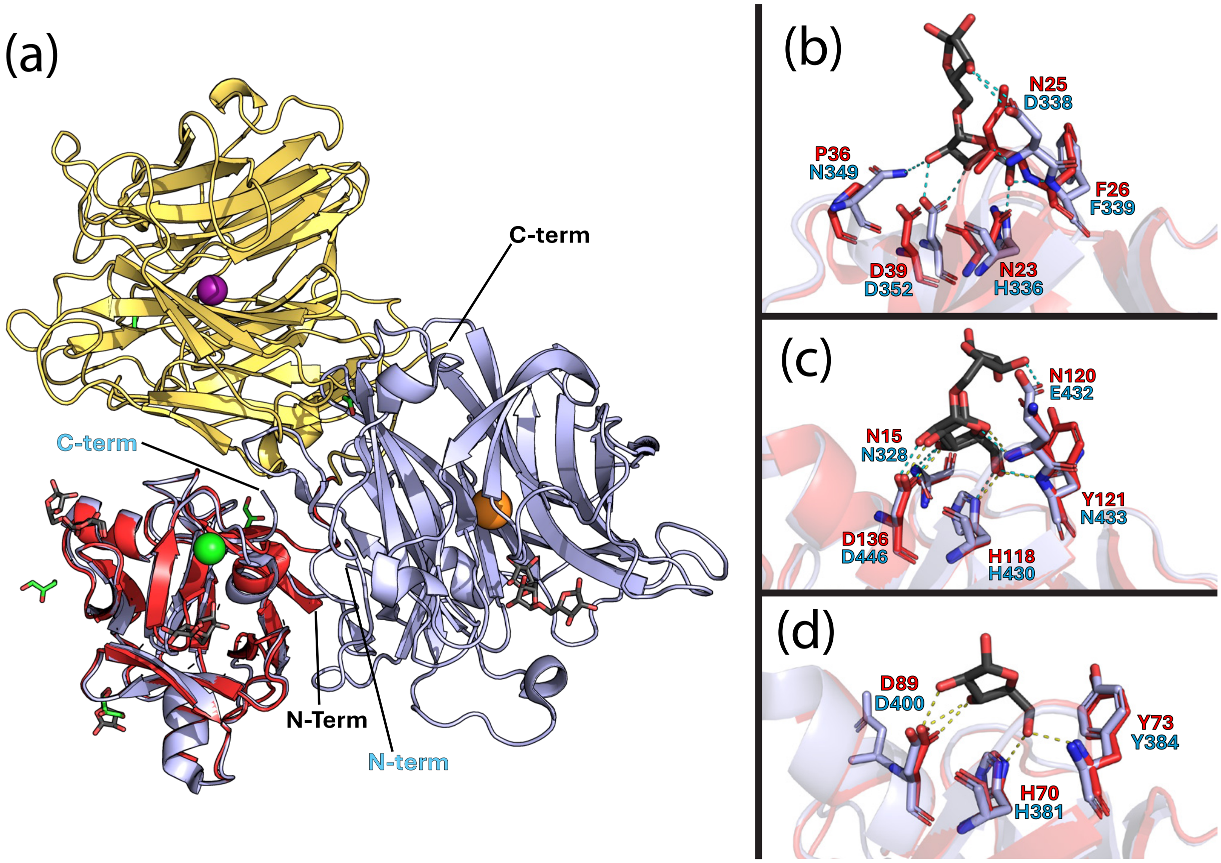

Fig S3. Mass spectra for AtAbf43C *in vitro* hydrolysis reactions on natural polysaccharides and oligosaccharides. **(a)** Table of retention times and visual key for relevant species. Reactions were performed without and with AtAbf43C on **(b)** arabinose, **(c)** xylose, **(d)** WAX, **(e)** BX, **(f)** SBA, **(g)** A2, **(h)** A3, **(i)** A4, **(j)** A5, **(k)** A2XXX, and **(l)** XA3XXX. For each reaction, a mass spectrum was acquired for a range of 8 to 18 min in retention time, as all products of interest have retention times within this range. All species were identified as $[M+Na]^+$ adducts.

(a)

Molecule	Formula	MW	(M+Na)+	Retention time of center of peak
Arabinose	C5H10O5	150.13	173.042	~15.85 min
Xylose	C5H10O5	150.13	173.042	~14.6 min
A2	C10H18O9	282.2	305.0843	~11.6 min
A3	C15H26O13	414.4	437.1266	~10.35 min
A4	C20H34O17	546.5	569.1688	~9.7 min
A5	C25H42O21	678.6	701.2111	~9.35 min
A-2-XXX	C20H34O17	546.5	569.1688	~9.9 min
X-A-3-XXX	C25H42O21	678.6	701.2111	~9.6 min

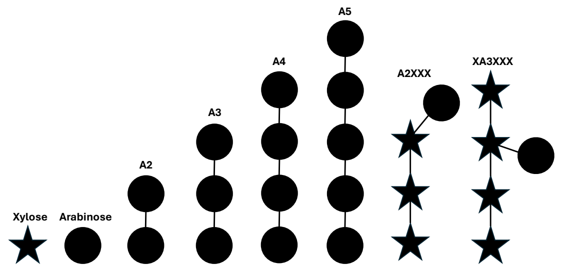

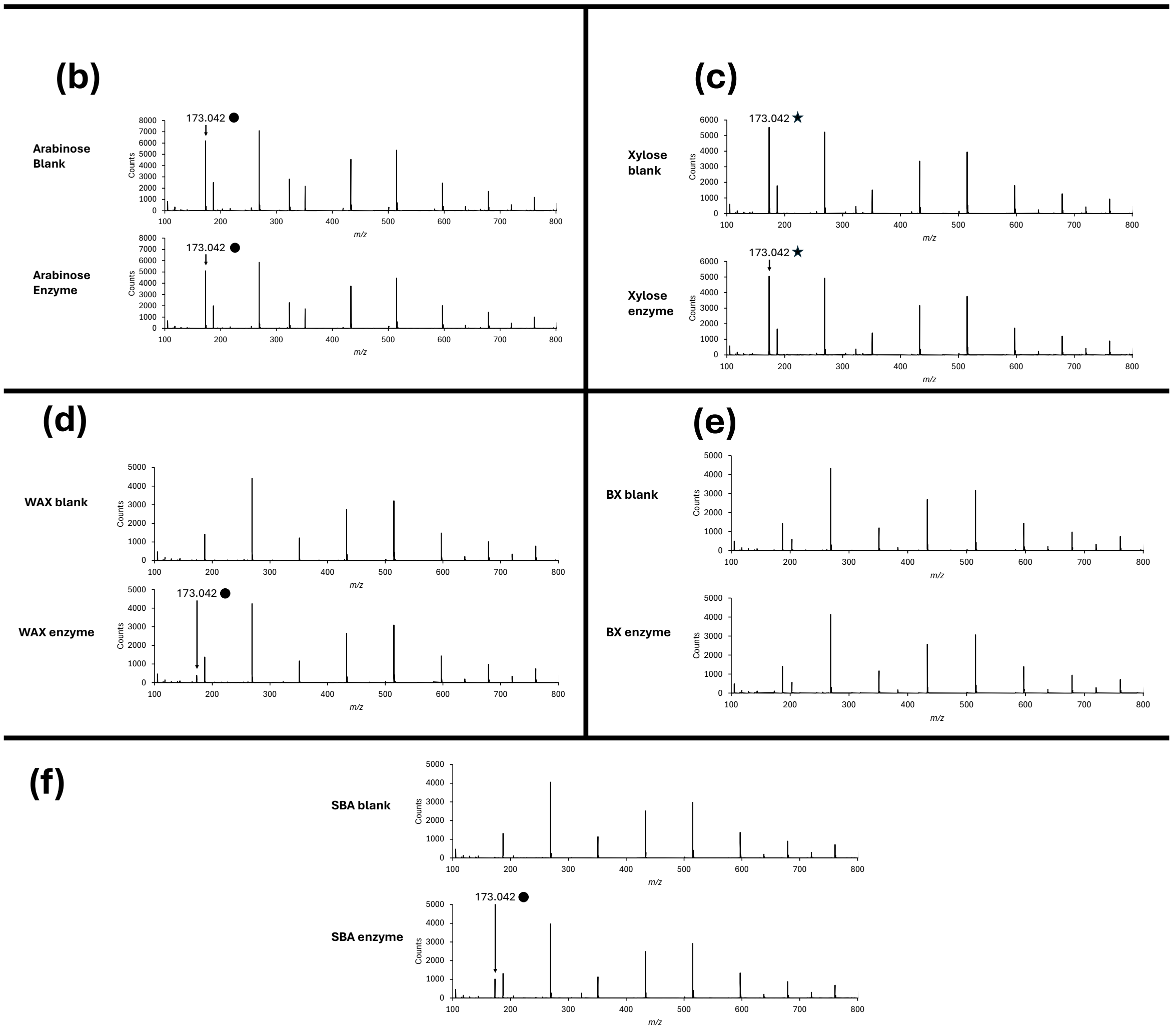

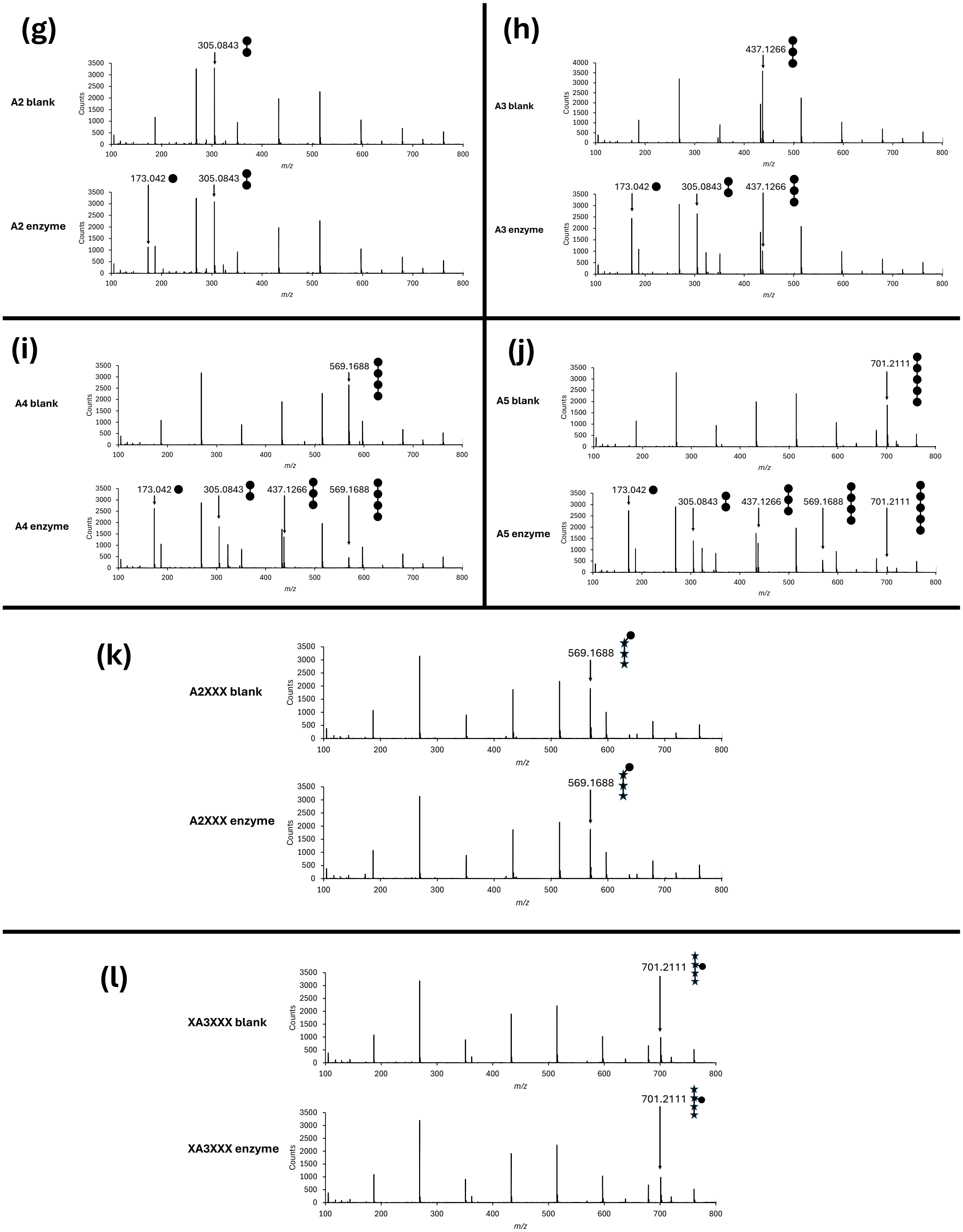
(g)
A2 blank
305.0843
A2 enzyme
173.042
305.0843
(h)
A3 blank
437.1266
A3 enzyme
173.042
305.0843
437.1266
(i)
A4 blank
569.1688
A4 enzyme
173.042
305.0843
437.1266
569.1688
(j)
A5 blank
701.2111
A5 enzyme
173.042
305.0843
437.1266
569.1688
701.2111
(k)
A2XXX blank
569.1688
A2XXX enzyme
569.1688
(l)
XA3XXX blank
701.2111
XA3XXX enzyme
701.2111
Counts
m/z
